# Organic and Synthetic Substitutes in Tracheal Reconstruction: A Scoping Review (2015–2025)

**DOI:** 10.3390/bioengineering12070704

**Published:** 2025-06-27

**Authors:** Ana Caroline dos Santos, Guilherme Machado Holzlsauer, João Paulo Ruiz Lucio de Lima Parra, Raí André Querino Candelária, Thamires Santos da Silva, Rodrigo da Silva Nunes Barreto, Maria Angelica Miglino

**Affiliations:** 1 Graduate Program in Anatomy of Domestic and Wild Animals, Faculty of Veterinary Medicine and Animal Science, University of São Paulo (FMVZ/USP), São Paulo 05508-270, Brazil; anacaroline.santos@usp.br (A.C.d.S.); raiandre@usp.br (R.A.Q.C.); 2 Department of Veterinary Clinic and Surgery, Faculty of Agricultural and Veterinary Sciences, São Paulo State University (UNESP), Jaboticabal 14884-900, Brazil; guilherme.holzlsauer@unesp.br; 3 Department of Animal Anatomy, University of Marilia (UNIMAR), Marília 17525-902, Brazil; joao.parra@unesp.br; 4 XenoBr, Rua do Matão, 106, Cidade Universitária, São Paulo 05508-090, Brazil; tssxenobr@gmail.com; 5 Department of Animal Morphology and Physiology, Faculty of Agricultural and Veterinary Sciences, São Paulo State University (UNESP), Jaboticabal 14884-900, Brazil; rodrigo.barreto@unesp.br; 6 Postgraduate Program in Structural and Functional Interactions in Rehabilitation, Postgraduate Department, University of Marilia (UNIMAR), Marilia 17525-902, Brazil; 7 Postgraduate Program in Animal Health, Production and Environment, University of Marilia (UNIMAR), Marilia 17525-902, Brazil

**Keywords:** transplant, trachea, tissue engineering, biocompatibility, tracheal reconstruction, scaffold

## Abstract

Tracheal defects have been the focus of research since the 19th century, but reconstructing this complex structure remains challenging. Identifying a safe, effective tracheal substitute is a key goal of surgery. This integrative review explores current tracheal substitutes and tissue engineering techniques. Data were collected from June 2024 to March 2025 from electronically available databases. Articles published between 2015 and 2025 were selected using the individualized protocol for each database. After screening 190 articles, 82 were excluded, and 108 were reviewed, with 100 meeting the final inclusion criteria. Recent substitutes include three-dimensional synthetic grafts made from polycaprolactone and copolyamide with thermoplastic elastomer, thermoplastic polyurethane and polylactic acid. Additionally, models using decellularized and recellularized tracheal matrix scaffolds and bioprinting techniques are being developed. Comparative studies of synthetic grafts and tracheal scaffolds, as well as cell self-aggregation methods to create tracheal analogues, are discussed. Advances in hybrid approaches combining synthetic polymers with extracellular matrix components aim to improve biocompatibility and functional integration. The importance of selecting appropriate preclinical animal models, such as goats, is also highlighted for translational relevance. Further research is required to refine protocols, overcome challenges related to vascularization and immune response, and ensure the development of clinically viable, long-lasting tracheal substitutes.

## 1. Introduction

The trachea is a component of the respiratory system responsible for connecting the larynx to the bronchi, thereby regulating airflow and facilitating breathing [1]. Due to its structural and functional complexity, tracheal reconstruction poses a significant clinical challenge [2,3]. According to the most recent Global Burden of Chronic Respiratory Diseases and Risk Factors report, more than four million people die annually as a result of chronic respiratory conditions [4]. Although pulmonary diseases are the primary contributors to respiratory system impairment in both humans and domestic animals, common tracheal pathologies include benign airway stenosis, tracheal or pulmonary tumors, localized defects, tracheal rupture, and post-extubation complications, such as tracheal collapse in German Dwarf Spitz dogs [5,6].

Conventional surgical approaches to treating acute tracheal damage include the use of artificial implants, allografts, and autologous grafts. However, these strategies often fail to restore full function and are associated with serious long-term complications [7]. In response to these limitations, the field of bioengineering, an interdisciplinary domain, seeks to develop biocompatible grafts capable of regenerating or repairing damaged tracheal tissue [8].

Recent advances in tissue engineering have enabled the fabrication of synthetic scaffolds using technologies such as three-dimensional (3D) bioprinting and electrospinning, in combination with polymers and hydrogels. Nevertheless, replicating the trachea’s specialized microenvironment remains a major challenge, primarily due to its complex extracellular matrix (ECM), which includes collagens, elastin, proteoglycans, and a dense vascular network [8,9]. Biological scaffolds derived from decellularized human or animal tissues have emerged as promising alternatives to conventional methods. These scaffolds retain the native ECM while eliminating cellular and immunogenic components, thereby minimizing the risk of rejection. Decellularization techniques, such as detergent-enzymatic membrane (DEM) treatment, laser microporation, and vacuum-assisted decellularization (VAD), have significantly enhanced scaffold quality [10]. Recellularization, using mesenchymal and epithelial stem cells, aims to restore native functionality. However, challenges such as achieving complete removal of cellular debris without compromising ECM integrity remain critical obstacles to the clinical translation of bioengineered tracheas [8,11].

This review provides a focused overview of recent advances in organic and synthetic tracheal substitutes, emphasizing 3D bioprinting, decellularization, and translational animal models. Unlike previous studies, it highlights improvements in graft biocompatibility and functionality, alongside the critical role of selecting anatomically relevant animal models for preclinical validation. Conventional reconstructive methods often face limitations such as structural failure and chronic inflammation. We hypothesize that bioengineered tracheal substitutes integrating decellularization, recellularization, and mechanical optimization can enhance tissue integration and reduce post-implantation complications. This study addresses key questions related to the development of innovative tracheal grafts, focusing on hybrid materials and advanced fabrication techniques. The use of large animal models, particularly goats, is emphasized due to their anatomical and biomechanical similarity to the human trachea, enhancing translational relevance. Overall, the integration of biological and mechanical strategies offers promising prospects for improving graft performance and clinical outcomes.

## 2. Materials and Methods

This systematic scoping review was conducted in accordance with the PRISMA-ScR guidelines [12], with the objective of mapping and synthesizing preclinical and experimental evidence on tracheal substitutes developed through bioengineering and tissue engineering techniques. The methodological design prioritized the identification of technological advances, relevant animal models, and challenges associated with the biocompatibility, functionality, and translational potential of these substitutes.

A comprehensive search strategy was implemented across seven electronic databases, PubMed/MEDLINE, Google Scholar, Virtual Health Library (VHL), LILACS, SciELO, the Virtual Library of Veterinary Medicine, and Semantic Scholar, covering the period from 2015 to 2025. To maximize sensitivity and specificity, both controlled vocabulary (MeSH/DeCS) and free-text terms were combined, adapted to the search syntax of each platform, and connected using Boolean operators. For example, the PubMed/MEDLINE search incorporated terms such as (“trachea” [Title/Abstract] OR “tracheal reconstruction” [Title/Abstract]) in combination with descriptors for tissue engineering techniques and animal models, while excluding review articles. In Latin American databases (LILACS and SciELO), terms were prioritized in Portuguese and Spanish, such as “engenharia de tecidos” and “reconstrucción traqueal”. Google Scholar utilized exact phrase searching and manual exclusion filters (e.g., “-review”) to reduce irrelevant results. The full list of search terms is available in Supplementary Materials.

The inclusion criteria were based on the PCC framework (Population, Concept, Context), as outlined in the JBI Manual for Scoping Reviews [13]. The population included animal models (e.g., rats, mice, rabbits, pigs, goats) and in vitro systems with preclinical relevance. The core concept focused on the development of tracheal substitutes using bioengineering and tissue engineering methods, including scaffolds, synthetic and natural frameworks, decellularized extracellular matrices, and 3D bioprinting technologies. Selected studies were required to evaluate at least one parameter related to biocompatibility, vascularization, mechanical properties, or tissue integration. The context was restricted to experimental and preclinical studies published between 2015 and 2025, with full text availability in English, Portuguese, or Spanish, indexed in recognized databases, and accessible through open access or institutional subscriptions (USP and UNESP). This time frame was selected to capture recent methodological innovations in regenerative medicine.

Exclusion criteria included secondary studies (e.g., systematic or narrative reviews), studies lacking empirical data, research not focused on the trachea, and studies unrelated to tissue bioengineering. These exclusions were intended to prevent duplication of previously synthesized findings and minimize interpretative bias, ensuring the analysis was grounded solely in primary experimental evidence. This approach strengthened the reliability of the synthesis and supported the identification of technical advances and emerging trends in tracheal substitute development (Table 1).

The study selection followed the PRISMA flowchart in three stages: identification, screening, and eligibility. In the initial phase, duplicate records were removed, and temporal filters were applied. During screening, two independent reviewers assessed titles and abstracts, achieving a Cohen’s Kappa coefficient of κ = 0.572. Discrepancies were resolved by a third reviewer to enhance methodological rigor. In the eligibility phase, full texts were assessed to confirm inclusion criteria.

Extracted data were compiled into a standardized matrix capturing study characteristics (author, year, country, animal model), intervention specifics (scaffold type and manufacturing method), and key findings (e.g., in vivo biocompatibility, mechanical performance). Qualitative synthesis categorized the results into three thematic areas: (i) materials and scaffold fabrication (e.g., hydrogels vs. synthetic polymers); (ii) vascularization challenges (e.g., use of growth factors, in vitro pre-vascularization); and (iii) limitations of animal models (e.g., anatomical discrepancies between species).

Several potential biases were considered in the study design and execution, in accordance with updated methodological recommendations [13]. These included publication bias, stemming from underreporting of negative or inconclusive results, as well as language bias, due to the inclusion criteria being restricted to English, Portuguese, and Spanish publications. Access bias was also acknowledged, as only full-text articles were included. Selection bias was mitigated through the dual-reviewer screening process and adjudication by a third reviewer.

Methodological heterogeneity among included studies, particularly regarding animal models and experimental protocols, was recognized as an intrinsic limitation. This was addressed through descriptive synthesis. All animal studies were assessed for ethical approval based on ARRIVE guidelines (Animal Research: Reporting of In Vivo Experiments) [14]. The review protocol was prospectively registered on the Open Science Framework. This comprehensive methodological strategy enabled not only the identification of research gaps but also the recognition of emerging trends, such as 4D bioprinting, the shift from traditional polymers to biomimetic hybrid structures, efforts toward protocol standardization, and the scalability of fabrication techniques. These insights are intended to inform future directions in tracheal regenerative medicine.

## 3. Results

**Figure 1:**
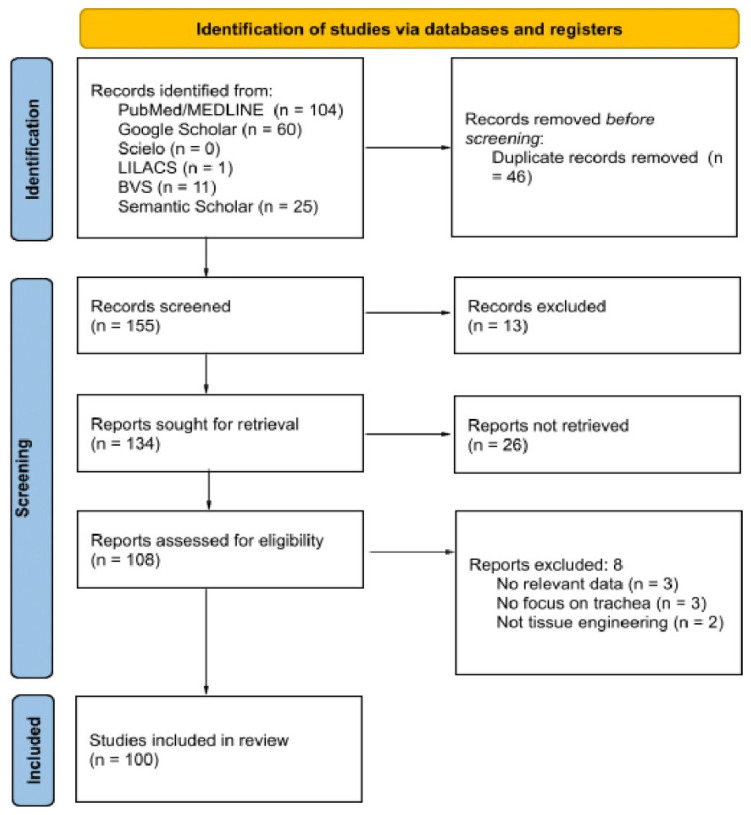
PRISMA flow diagram used for the systematic review.

According to the PRISMA flow diagram (Figure 1), this scoping review included 100 articles that met the established eligibility criteria. These studies were conducted across various countries, reflecting broad international interest in the development of natural and synthetic tracheal substitutes for transplantation. A diverse range of materials, both natural and synthetic, were used with the goal of enhancing functionality and biocompatibility in tracheal replacement strategies (Table 2). The following is a summary of the main types of materials identified in the included studies, along with their respective frequencies.

### 3.1. Studies Evaluating Synthetic and Natural Materials In Vitro

In vitro studies have investigated a variety of materials for tracheal transplantation, emphasizing both synthetic and natural scaffolds. Decellularized extracellular matrix (dECM) scaffolds derived from the tracheas of species such as pigs and rabbits have shown promise due to their ability to preserve the native architecture of the tissue, offering an optimal environment for cell culture [15,16,17]. These matrices facilitate assessments of cell viability, adhesion, and differentiation, critical parameters for modeling functional tracheal tissue prior to transplantation. In parallel, synthetic scaffolds such as poly(ε-caprolactone) (PCL) have gained attention for their versatility, biocompatibility, and suitability for fabrication using techniques such as electrospinning and 3D printing [18]. Additional synthetic materials include chitosan and thermosensitive elastomers (PUU-POSS), which are designed to replicate the trachea’s biomechanical properties [19].

Hybrid scaffolds, which integrate synthetic polymers with natural biomaterials, such as collagen, aim to improve tissue regeneration by enhancing mechanical and biological performance. Examples include PCL-PEG bioinspired laryngotracheal patches loaded with dexamethasone to reduce inflammation and promote regeneration. These designs seek to optimize healing and bring engineered substitutes closer to the requirements of successful clinical application (Table 3).

### 3.2. Studies Evaluating Synthetic and Natural Materials In Vivo

Tracheal substitutes evaluated in vivo are generally categorized as synthetic, decellularized, or bioprinted. Each category presents distinct advantages in terms of mechanical properties, biocompatibility, and clinical applicability. Decellularized matrices derived from animal tissues, particularly from rabbits and pigs, have been extensively studied for their capacity to support tissue regeneration [25]. Some matrices are further enhanced with agents such as genipin to improve structural stability and cell integration [11,23,26,27,28]. Collagen-based membranes and mucosal matrices from porcine sources have also been investigated for their bioactive potential [26,27,29].

Synthetic scaffolds, including those made from PCL, PLA, PLGA, and PU, are valued for their manufacturing precision and mechanical durability. These polymers are often combined with hydrogels such as collagen, alginate, or silica to improve their biological performance [30,31,32,33,34]. 3D printing with PCL has facilitated the development of patient-specific implants, improving anatomical fit and clinical outcomes [35,36,37,38,39]. PET/PU scaffolds are another relevant material used for structural support and in the fabrication of stents [40,41].

3D bioprinting is a cutting-edge technique that allows the layer-by-layer construction of tracheal scaffolds using cells, biomaterials, and bioactive factors. This technology enables the fabrication of hybrid scaffolds combining synthetic polymers, such as PCL, with natural materials such as silk fibroin and hydrogels. The use of bioinks containing bone marrow-derived stem cells (BMSCs), adipose-derived stem cells, or human mesenchymal stem cells (hTMSCs), along with components such as collagen and alginate, has further improved graft functionality [42,43,44,45,46,47,48,49].

Customization of scaffold geometry using 3D printing enhances anatomical compatibility and integration, reducing the risk of complications. However, challenges persist in mimicking the rigidity and elasticity of native tissue, achieving sufficient vascularization and epithelialization, and avoiding adverse immune responses. Reported complications include implant extrusion, immune rejection, lack of host integration, and infections [19,20,21,22,23,24,25,34,35,36,37,38,39,40].

Despite these limitations, 3D bioprinting remains a highly promising avenue for future reconstructive tracheal surgery. Innovations under exploration include the use of pre-vascularized scaffolds, dynamic bioreactors, 4D printing with stimuli-responsive materials, and novel bioinks. These strategies aim to enhance graft performance and safety, bringing the field closer to a functional and clinically viable tracheal substitute.

Hybrid scaffolds, which blend synthetic and natural materials, represent a notable innovation. Bilayer or multilayer designs, such as PLLA-CL tubes with distinct inner and outer layer compositions, support both epithelial adhesion and cartilage regeneration. Other approaches use 3D-printed scaffolds incorporating PCL modified with hydroxyapatite (HA) and embedded hydrogels to improve cellular integration. However, manufacturing these hybrid structures remains technically complex and requires precise integration of materials. The limited availability of in vivo transplant studies and long-term follow-up data remains a barrier to clinical translation.

### 3.3. Characterization and Analysis of Biomaterials

In studies involving biomaterials for tracheal transplantation in animal models, various analytical techniques were employed to assess structural, mechanical, cellular, and biochemical characteristics before and after implantation. Post-implant tracheoscopy enabled direct visualization of graft integration and detection of rejection or adaptation. Histological analysis of stained tissue sections provided insights into epithelial and cartilage regeneration [23,25,47,49,50,51,52,53,54].

Scanning electron microscopy (SEM) was widely used to evaluate scaffold porosity, surface quality, and compliance with design specifications, which are crucial for cell attachment and proliferation [27,40,46,55,56,57,58,59,60]. Micro-computed tomography (micro-CT) provided detailed 3D imaging of pore architecture and density, contributing to assessments of tissue compatibility and implant viability [61,62,63].

Mechanical testing, including compression, bending, and tensile strength measurements, was conducted to determine scaffold durability under physiological stress. These tests ensured that implants could withstand respiratory forces without compromising airway patency [4,25,27,41,54,58,60,64,65,66,67,68,69].

Cell viability assays, such as in vitro cultures, were performed to evaluate the scaffold’s support for cell growth and differentiation. DNA quantification [23,32,40,41,46,54,56,60,61,69,70,71] and collagen content analysis [23,29,39,46,49,50,54,57,61,69,70,72] offered additional data on extracellular matrix formation and scaffold-tissue interactions. Immunohistochemistry (IHC) was used to detect specific markers, such as type II collagen and cytokeratin, indicating epithelial and chondrogenic differentiation [73,74,75].

Some studies also monitored the release of anti-inflammatory agents such as dexamethasone and quercetin [21,30], evaluating their roles in modulating inflammation and supporting tissue regeneration. These analyses provided a comprehensive understanding of biomaterial performance and their therapeutic potential in restoring respiratory function.

### 3.4. Biocompatibility Analysis

Several approaches demonstrated promising outcomes for tracheal regeneration, with a focus on various scaffolds and decellularized matrices. Notably, biosheets composed of collagenous tissue supported complete epithelial and vascular regeneration within three months, with sustained respiratory function and 100% animal survival at nine months, highlighting their high biocompatibility and long-term repair potential [50].

Another promising material was the PET/PU 2:8 scaffold, produced via coextrusion and electrospinning. It exhibited mechanical properties similar to the native sheep trachea and promoted effective cell adhesion and tissue regeneration [56,76]. PCL-based scaffolds, especially when combined with collagen hydrogels or stem cells, consistently demonstrated favorable outcomes in terms of biocompatibility, structural support, and epithelial-cartilage regeneration [19,27,32,46,54,64,66,77,78].

3D-printed PCL scaffolds seeded with autologous cells promoted robust tissue regeneration and airway patency, indicating potential for clinical application [39,47,58,78,79]. The combination of PCL and collagen enhanced scaffold integration and tissue formation. Among the most innovative constructs, tissue-engineered tracheas (TETs) supported by 3D-printed PCL and autologous chondrocytes achieved high survival rates and minimal stenosis, with histological evidence of functional tissue regeneration [80].

Decellularized tracheal matrix scaffolds treated with genipin and seeded with BMSCs also showed improved tissue regeneration and cell survival, underscoring the value of combining biological scaffolds with autologous cells.

### 3.5. Limitations and Gaps

Despite notable advances, several limitations persist. Scaffolds made from synthetic polymers, such as PCL, collagen hydrogels, PET/PU, and PLA, exhibited good biocompatibility and regenerative potential [20,46,47,54,56,80,81]. However, moderate inflammatory responses were observed in some models, particularly those involving laser-modified PCL or hydrogel coatings [52,63,80].

Other challenges included excessive granulation tissue formation, incomplete epithelialization, and suboptimal integration, especially in models using PLA stents or modified silk fibroin matrices [41,82,83]. While collagen-based scaffolds generally integrated well with host tissue [53,84], hybrid scaffolds incorporating PCL and gelatin sometimes failed to support adequate epithelial regeneration [46].

Studies involving decellularized tissues and 3D-printed PCL scaffolds often showed promising results in terms of integration and regenerative support [23,26,27,85]. Nonetheless, issues such as excessive inflammation and poor revascularization were noted in constructs lacking cellular or vascular support elements [76,86,87]. These findings highlight the importance of incorporating pre-vascularization strategies or stem cells to enhance graft success and reduce complications such as necrosis [52,88].

Long-term biocompatibility also remains a concern, particularly in allogeneic and xenogeneic grafts, where immune rejection and fibrosis have been reported [45,68,81,83,89,90]. Scaffold architecture must be carefully controlled to avoid stenosis or fibrotic obstruction [91].

Emerging strategies combining scaffolds with bioactive agents, such as TGF-β3, aim to mitigate inflammation and enhance tissue integration [43]. While these approaches are promising, further research is needed to address persistent challenges such as structural defects, stenosis, necrosis, and mucus obstruction [37,45,82]. Effective modulation of inflammation, integration with host tissues, and cartilage regeneration remain critical to advancing safe and functional tracheal substitutes.

## 4. Discussion

Tissue bioengineering is an interdisciplinary field that combines principles of biology, engineering and medicine to create functional tissues and organs in the laboratory, with the aim of replacing or repairing damaged or diseased tissues. This field has the potential to revolutionize regenerative medicine by offering solutions to medical problems that currently have no effective treatments. Tissue engineering uses biocompatible materials such as scaffolds or polymers and involves the design and construction of three-dimensional structures that simulate the physical and functional properties of biological tissues. These structures incorporate cells, growth factors and other biomolecules that are important for tissue regeneration. In order for tissue bioengineering to advance in tracheal reconstruction and enable more effective clinical applications, it is necessary for the experimental models used to reproduce human biomechanical conditions more faithfully, ensuring that the results obtained in the laboratory can be translated into medical practice.

### 4.1. The Role and Limitations of Animal Models in Tracheal Bioengineering

Despite the growing body of literature demonstrating successful tracheal replacements in murine and lagomorph models, such as rats and rabbits [69], the direct applicability of these findings to the human clinical setting may be limited by fundamental differences in cervical biomechanics. As demonstrated by [92], the kinematics of the head and cervical spine in rats involves a combination of movements at the craniocervical and cervicothoracic junctions, contrasting with the predominance of rotation at the cervicothoracic junction observed in humans. This disparity in movement patterns can significantly influence the mechanical stresses imposed on an implanted tracheal substitute, potentially affecting its long-term integration and functionality. In this context, we propose that the goat (Capra aegagrus) represents a more translationally relevant animal model for the study of tracheal transplants. The goat was considered by [93] to be the most suitable animal model for tracheal surgery due to its human-like cervical anatomy, easy access to the operation site and easy handling.

**Figure 2:**
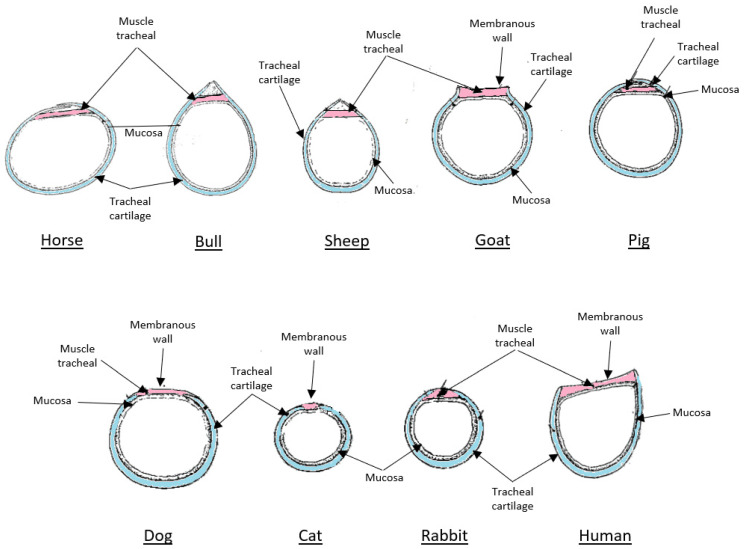
Schematic representation of the tracheal rings of different species (horse, ox, cat, dog, goat, human, rabbit, pig and sheep), highlighting anatomical variations in the shape of the tracheal cartilage and membranaceous wall. These differences influence respiratory dynamics and have implications for intubation, tracheostomy and the bioengineering of tracheal substitutes.

In addition, only the goat shows the same tracheal structure as humans and is, therefore, considered the ideal model. While in horses and pigs the tracheal ring portions overlap, and the space between them is filled by the tracheal membrane and muscles, as well as the tracheal mucosa, in cattle, sheep and rabbits the tracheal ring portions come together to form a ridge. Even so, there is a small opening between the two portions of the cartilage, a space also closed off by the tracheal membrane and muscle, as well as the tracheal mucosa. Only in dogs and goats do the parts of the tracheal rings not join together. The space corresponding to a quarter of the tracheal ring in the dog is completed by the membrane, tracheal muscle and tracheal mucosa internally. The tracheal structure and architecture most similar to that of humans is that of the goat (Figure 2). In this case, the shape of the tracheal ring resembles a “lyre”, with the space between the two portions of the ring corresponding to one-third of its circumference. This space is filled by the tracheal membrane, tracheal muscle and mucosa [94]. Therefore, the anatomy of the caprine trachea, in terms of size and structure, more closely resembles that of humans, and, crucially, its cervical movements exhibit greater similarity to the flexion and extension pattern prevalent in the human species, making it an ideal model for assessing the efficacy and durability of new tracheal replacement therapies in a more pertinent biomechanical context.

### 4.2. Inherent Challenges in Translating Preclinical Findings to Clinical Practice

However, even with improved anatomical and biomechanical fidelity offered by models such as the goat, significant fundamental limitations inherent to preclinical research persist, critically hindering the translation of promising findings into reliable clinical therapies. Beyond the specific biomechanical differences discussed earlier [92], crucial discrepancies remain in tracheal mechanics and healing dynamics across species. Humans possess powerful cough reflexes, generating high transient pressures and shear forces [95] often unmatched in animal models under experimental conditions. Daily activities, such as speech and varying exertion levels, impose unique cyclic loads not fully replicated. Furthermore, mechanical properties may not scale linearly from animal dimensions to the adult human trachea, affecting predictions of long-term stability and fatigue resistance [95]. Biologically, healing rates, inflammatory patterns, and chondrogenesis efficiency can differ substantially [9], potentially masking issues such as chronic rejection or improper scaffold degradation timing that would only manifest over longer human timeframes. Critically, subtle immunological differences mean that materials deemed biocompatible in animals might elicit unforeseen adverse immune responses or long-term foreign body reactions in humans. These factors collectively limit the predictive power of animal studies, irrespective of the chosen species, impacting data interpretation for clinical translation.

Compounding these issues is the significant gap between simplified in vitro assessments and the complex in vivo reality. While in vitro tests provide valuable initial screening for cytotoxicity or cell–scaffold interactions [15,18,19,22], they fail to capture the following key physiological challenges essential for clinical success.

Vascularization: This remains a primary bottleneck for construct survival and integration. Rapid host blood vessel infiltration is vital in vivo to prevent ischemia, central necrosis, and implant failure, especially in larger or thicker constructs [52,88], a factor inherently absent in vitro. Achieving robust, stable, and functional vascular networks reliably within engineered tracheas remains largely unresolved.

Immune Response and In Vivo Biocompatibility: The in vivo environment presents complex immunological challenges. Materials deemed safe in vitro can trigger acute or chronic inflammation, fibrosis leading to stenosis, or specific adaptive immune rejection in vivo, particularly with allogeneic/xenogeneic materials or cells [28,45,68,81,83,89]. Predicting the long-term interplay between the implant, its degradation products, and the host immune system is exceptionally difficult based on in vitro data alone.

Long-Term Stability and Remodeling: Constructs in vivo face continuous mechanical stresses (breathing, coughing, movement) and a dynamic biochemical environment (enzymatic degradation), which are rarely accurately simulated long term in vitro. This creates uncertainty regarding long-term structural integrity. Scaffolds must balance providing temporary support with appropriate degradation and replacement by host tissue, avoiding premature mechanical collapse or excessive persistence that hinders tissue remodeling and may act as a nidus for infection [37,41,82].

Functional Integration: Beyond mere structural replacement, achieving true functional restoration, including effective mucociliary clearance, appropriate cartilage stiffness/flexibility, and potentially sensory innervation, is essential for clinical success but largely untestable in vitro and represents a higher bar that current engineered tracheas often fail to meet in vivo.

Overcoming these intertwined challenges related to preclinical model limitations, vascularization, immunogenicity, long-term mechanobiological stability, and functional integration is paramount for advancing tracheal substitutes towards safe and effective clinical application.

### 4.3. Hybrid Approaches in Tracheal Reconstruction

The correlation between the choice of animal model and the development of hybrid tracheal reconstruction methods is important for the translational validation and clinical applicability of these approaches. Tracheal reconstruction using hybrid methods, which combine organic and synthetic components, is being explored to improve the biocompatibility and functionality of the implants. Several studies report the use of different combinations of biomaterials with the aim of combining the mechanical strength of synthetic polymers with the bioactivity of natural components.

Hybrid biodegradable polymer and extracellular matrix (ECM) scaffolds have been used by [41,47,48], who described the use of synthetic polymers such as poly(ε-caprolactone) (PCL), poly(lactic-co-glycolic) (PLGA) and polyurethane (PU) combined with ECM derived from natural tissues. The addition of ECM aims to provide biochemical signals that facilitate cell adhesion and proliferation, improving tissue remodeling. These materials show reduced inflammatory response and greater cell integration when compared to purely synthetic scaffolds. Another strategy involves the use of nanocomposites coated with ECM or collagen. The authors [38,75] described the incorporation of ECM or type I collagen on the surface of synthetic scaffolds, improving cell adhesion and reducing the adverse immune response. Synthetic tracheal prostheses with autologous cells have also been explored, wherein authors [48,53] developed silicone or polyurethane prostheses associated with the cultivation of the patient’s own cells on the surface of the implant.

Synthetic materials were developed by [30,52,75,96,97] to create tracheal substitutes and evaluate their biocompatibility in vitro and in vivo. In a recent study, ref. [75] designed a two-layer tubular scaffold combined with cells derived from human induced pluripotent stem cells (iPSCs). The artificial trachea was manufactured by combining PCL nanofibers (inner layer) with 3D-printed PCL microfibers (outer layer). To maximize the regeneration of the tracheal mucosa and cartilage in vivo, human bronchial epithelial cells (hBECs), iPSC-derived mesenchymal stem cells (iPSC-MSCs) and iPSC-derived chondrocytes (iPSC-Chds) were used. Similar work by [44,98] used synthetic biopolymers in combination with mesenchymal and epithelial stem cells to create functional tracheal substitutes.

### 4.4. Advanced Fabrication Using 3D Printing and Bioprinting

3D printing of structures with biomaterials and cells is another hybrid method that has been studied. Considering the tracheal reconstruction in Regenerative Medicine, biomaterial sources are indispensable in regenerating anatomical or functional damaged sites [99]. Key parameters such as mechanical strength, porosity, and biodegradability are critical [100,101]. However, most available biomaterials suit only stiffness, jeopardizing functionality [102,103], whereas cell-laden structures fabricated with 3D-bioprinting have shown outstanding results [104].

3D bioprinting presents a promising alternative by enabling the fabrication of specific tracheal constructs using biomaterials designed to mimic the natural extracellular matrix (ECM) [105]. The main techniques include extrusion, laser-assisted, and inkjet bioprinting, each with specific requirements for bio-ink viscosity and its own set of advantages and limitations regarding resolution, cell survival, and cost [106,107,108,109,110,111,112,113,114]. The formulation of bio-inks is crucial, often using natural or synthetic polymers, such as gelatin, alginate, PCL, and PLGA [71,107,108,109,110,111,112,113,114,115,116,117,118,119]. However, a primary challenge remains in combining high-resolution printing with an environment that promotes cell adaptation and survival [120]. Consequently, hybrid or composite materials, such as hydrogels mixed with decellularized extracellular matrices (dECM), are emerging as suitable alternatives to achieve both mechanical stability and bioactivity [121,122,123,124]. Recent advances also explore 4D bioprinting and patient-specific designs derived from medical imaging [51,124,125,126]. Several studies have demonstrated the utility of 3D printing, such as the work by [97] who designed a PCL structure combined with a PLA cylinder in rabbits.

### 4.5. The Role of Decellularized Matrices as a Biological Scaffold

Decellularized tracheal matrices have been evaluated by [28,127,128] for biocompatibility and potential as substitutes for extensive defects. In a study carried out in the United States, ref. [129] confirmed the biocompatibility of recellularized canine tracheal scaffolds, preserving their structure and glycoproteins after chemical–physical decellularization. These scaffolds were implanted subcutaneously in mice. Studies have shown that retaining a portion of the native cartilage through partial decellularization can improve mechanical resistance [28,130]. Other approaches have used decellularized matrices from different sources, such as bovine carotid arteries [131] or small intestinal submucosa [132], to repair tracheal defects in various animal models. Furthermore, ref. [128] used orthotopically transplanted bioengineered tracheas in ferrets to track the contribution of fluorescent stem cells. As an alternative, ref. [17] developed a scaffold-free tracheal analog by self-aggregating cartilaginous rings in vitro.

### 4.6. Bioengineering Strategies

The studies analyzed show that the combination of synthetic biomaterials and biological components can result in implants that are more functional and well tolerated by the body. The results of [27] suggest that 3D-printed tracheal grafts have better biomechanical properties than decellularized tracheal grafts. However, decellularized tracheal grafts showed better biocompatibility. These findings are in line with those of [129], who demonstrated tissue preservation and proliferation in vivo with decellularized tracheal implants. The biocompatibility observed in the different methods varied according to the material used, with those containing ECM or collagen showing greater cell adhesion and a lower inflammatory response. However, challenges still remain, such as the need to optimize manufacturing processes, the durability of the materials and their ability to integrate with the host tissue in the long term.

## 5. Conclusions

Tissue bioengineering has advanced in the development of tracheal substitutes by combining synthetic biomaterials and biological components to optimize implant functionality. The selection of an appropriate animal model, such as the goat, is crucial for translational validation due to its anatomical similarity to the human trachea. Strategies such as hybrid scaffolds, 3D printing, and decellularized matrices have demonstrated potential in enhancing implant biocompatibility and durability. The combination of synthetic polymers with ECM or collagen has been shown to reduce inflammatory responses and improve cellular adhesion, while recellularization techniques promote functional regeneration of the tracheal mucosa.

However, challenges such as manufacturing optimization, mechanical stability, and economic feasibility remain. Future research should focus on refining protocols and scaling up methodologies for clinical application. Tracheal bioengineering continues to evolve, bringing the field closer to viable alternatives for human tracheal reconstruction.

## Figures and Tables

**Table 1:** Methodological framework of the scoping review.

Stage	Description
Review Type	Scoping review following the PRISMA-ScR protocol.
Databases	PubMed, Google Scholar, VHL, LILACS, SciELO, VETLib, Semantic Scholar.
Timeframe	2015–2025
Inclusion Criteria	Experimental studies with animal models or in vitro, focused on tracheal substitutes and bioengineering.
Exclusion Criteria	Reviews, studies without empirical data, or unrelated to the trachea.
Selection Process	PRISMA screening (duplicate removal, evaluation by two reviewers, full-text reading).
Data Extraction	Organized into a matrix (author, year, animal model, scaffold, method, results).
Bias Analysis	Consideration of publication bias, language bias, and methodological heterogeneity.
Registration	Protocol registered in PROSPERO.

**Table 2:** Comparative characteristics of tracheal scaffold types.

Scaffold Type	Example Biomaterials	Fabrication Methods	Biomechanical Properties (Advantages/Limitations)	Observed Biocompatibility	Vascularization or Cellular Integration	Animal Model Used
Synthetic	Poly(caprolactone) (PCL)([-O-(CH2)5-CO-]n), polyurethane ([-OC-NH-R-NH-CO-O-R’-O-]n), thermoplastic elastomers (PCU), POSS-PCU, PET/PU ([-O-CH2-CH2-O-CO-C6H4-CO-]n)	3D printing, electrospinning, injection molding	Advantages: High dimensional customization; strong mechanical resistance. Limitations: Excessive stiffness, risk of granulation tissue formation, variable foreign body reaction.	Moderate to low; depends on surface treatment and chemical composition. May trigger chronic inflammation.	Requires angiogenic factor modification or stem cell addition to improve integration. Poor intrinsic vascularization.	Rabbits, rats, sheep
Decellularized	ECM from porcine, canine, rabbit tracheas; bladder/mucosa matrix	Chemical (SDS, Triton X-100, SLES), enzymatic (DNase, RNase), physical (ultrasound, freeze–thaw cycles, agitation)	Advantages: Preserves natural structure; retains native ECM cues. Limitations: Structural weakening risk; possible immunogenicity if incomplete decellularization.	High with complete DNA/antigen removal. Lower immune risk when combined with DNase/RNase.	Good integration when pre-seeded with mesenchymal or epithelial cells. Vascularization dependent on implantation strategy.	Rabbits, mice, dogs, ferrets, pig
Hybrid	Hydrogels (gelMA, collagen, alginate), PCL-hydrogel combinations with embedded cells	Extrusion bioprinting, inkjet, SLA, DLP, FDM	Advantages: Highly customizable; supports native-like cell distribution. Limitations: Lower initial mechanical strength; long-term viability challenges.	Excellent; live cells improve biocompatibility. Low immune rejection with autologous/immunoprivileged cells.	High cellular integration potential; vascularization possible with suitable bioinks or angiogenic strategies.	Rabbits, ferrets

**Table 3:** In vitro tests for the evaluation of biomaterials in tracheal reconstruction.

Source	Tracheal Substitute	Biomaterial Characterization	Relevant Results	Biocompatibility Findings
[20]	Hybrid scaffolds of thermoresponsive elastomer (PUU-POSS) and type I collagen, fabricated via TIPS and 3D printing	SEM; Confocal microscopy: collagen distribution; Mechanical analysis; Contact angle testing: surface hydrophilicity; XPS; quantification of structural characteristics.	3D-TIPS scaffolds with collagen improved hydrophilicity, supported epithelial differentiation, and formed a functional barrier with high TEER in coculture.	Scaffolds supported cell adhesion, proliferation, and differentiation, while coculture promoted a mature epithelium with tight junctions, barrier function, and mucus production, indicating potential for mucociliary function.
[19]	Electrospun nanofibers of poly(ε-caprolactone) (PCL) and depolymerized chitosan.	SEM, XRD, FTIR, tensile testing, contact angle analysis.	Successful nanofiber fabrication; chitosan influenced mechanical properties and hydrophilicity.	Good cell adhesion, no cytotoxicity, and maintained cell morphology.
[21]	Bioinspired laryngotracheal patch with PCL-PEG and dexamethasone release.	SEM, laser profilometry, mechanical testing, mucoadhesion, cytotoxicity.	Higher PCL particle density enhanced strength and adhesion.	Biocompatible and promising for tracheal applications.
[22]	Decellularized ECMs from porcine trachea and bladder.	DNA quantification, histology, immunohistochemistry, protein quantification.	HBECs exhibited better growth and differentiation on porcine tracheal ECM.	Both ECMs were biocompatible, but the tracheal ECM promoted superior differentiation.
[16]	Decellularized extracellular matrix (dECM) from porcine trachea	DNA quantification, histology, structural analysis	Efficient decellularization (>95% DNA removal) with preserved extracellular matrix	Good matrix preservation, indicating potential for biocompatibility.
[18]	Decellularized hybrid grafts biofabricated in 3D (PCL + gamma radiation)	Compressive tesing, histology	Decellularized scaffolds showed lower compressive strength; gamma radiation affected mechanical properties	Histology confirmed cell removal; the combination of 3D bioprinting and decellularized ECM is promising
[15]	Decellularized rabbit tracheal scaffold	DNA analysis, biochemical assays, histology, polarized microscopy	Effective protocol for cell removal while preserving ECM; protocol variations affected ECM integrity	Decellularization removes immunogenic components while preserving ECM; potential for clinical use
[23]	Decellularized rabbit tracheal biomatrix	Histology, DNA and GAG quantification, SEM, and biomechanical tests	Decellularization removed cellular components while preserving structure and ECM; slight reduction in tensile strength	Potential as a biocompatible scaffold; in vivo studies needed for confirmation
[24]	Porcine tracheas subjected to different decellularization methods, including supercritical carbon dioxide (scCO_2_)	Biomaterial characterization after decellularization	Decellularization and sterilization with scCO_2_ preserved the extracellular matrix, glycosaminoglycans (GAGs), and collagen structure, maintaining suitable mechanical	Scaffolds supported cell adhesion, proliferation, and viability, with efficient DNA removal and GAG retention, indicating a promising method for biomaterials

## Data Availability

Data is contained within the article or Supplementary Material.

## Supplementary Materials

The following supporting information can be downloaded at: https://www.mdpi.com/article/10.3390/bioengineering12070704/s1. Refs. [12,133].
